# Methodological design for the assessment of physical activity and sedentary time in eight Latin American countries - The ELANS study

**DOI:** 10.1016/j.mex.2020.100843

**Published:** 2020-02-25

**Authors:** Gerson Luis de Moraes Ferrari, Irina Kovalskys, Mauro Fisberg, Georgina Gómez, Attilio Rigotti, Lilia Yadira Cortés Sanabria, Martha Cecilia Yépez García, Rossina Gabriella Pareja Torres, Marianella Herrera-Cuenca, Ioná Zalcman Zimberg, Viviana Guajardo, Michael Pratt, Shaun Scholes, Priscila Bezerra Gonçalves, Dirceu Solé, ELANS Study Group

**Affiliations:** a Centro de Investigación en Fisiologia del Ejercicio – CIFE, Universidad Mayor, Av. Sánchez Fontecilla #13.010, Campus Estadio Mayor, Peñalolén, Santiago, Chile; b Departamento de Pediatria da Universidade Federal de São Paulo, São Paulo, Brazil; c Commitee of Nutrition and Wellbeing, International Life Science Institute (ILSI-Argentina), Buenos Aires, Argentina; d Instituto Pensi, Fundação José Luiz Egydio Setubal, Hospital Infantil Sabará, São Paulo, Brazil; e Departamento de Bioquímica, Escuela de Medicina, Universidad de Costa Rica, San José, Costa Rica; f Departamento de Nutrición, Diabetes y Metabolismo, Escuela de Medicina, Centro de Nutrición Molecular y Enfermedades Crónicas, Pontificia Universidad Católica, Santiago, Chile; g Departamento de Nutrición y Bioquímica, Pontificia Universidad Javeriana, Bogotá, Colombia; h Colégio de Ciencias de la Salud, Universidad San Francisco de Quito, Quito, Ecuador; i Instituto de Investigación Nutricional, La Molina, Lima, Peru; j Centro de Estudios del Desarrollo, Universidad Central de Venezuela (CENDES-UCV)/Fundación Bengoa, Caracas, Venezuela; k Departamento de Psicobiologia, Universidade Federal de São Paulo, São Paulo, Brazil; l Institute for Public Health, University of California San Diego, La Jolla, CA, USA; m Institute of Epidemiology and Health Care, University College London, London, United Kingdom; n Programa de Pós-Graduação em Tecnologia em Saúde, Pontifícia Universidade Católica do Paraná, Paraná, Brazil; o Grupo de Pesquisa em Atividade Física e Qualidade de Vida, Pontifícia Universidade Católica do Paraná, Paraná, Brazil

**Keywords:** Epidemiology, Physical activity, Sedentary behaviour, Accelerometry, Public health, Latin America

## Abstract

Worldwide studies of physical activity and sedentary time have historically under-represented low- and middle-income countries due to the lack of surveillance data. The purpose of this paper is to describe the methods and procedures used for the assessment of physical activity and sedentary time in the Latin American Study of Nutrition and Health (*Estudio Latinoamericano de Nutrición y Salud*; ELANS). ELANS is a multicentre, cross-sectional and surveillance study of a nationally representative sample from eight Latin American countries: Argentina, Brazil, Chile, Colombia, Costa Rica, Ecuador, Peru, and Venezuela. Two instruments were used to evaluate different domains and intensities of physical activity and sedentary time: self-reported data and a triaxial accelerometer (model GT3X+). ELANS will generate important self-reported and objective information for the Latin American populations, namely:

• evidence on the distribution of physical activity and sedentary time across population subgroups (e.g. sex, age, socioeconomic- and educational level). These sets of information will increase the evidence base and can help to inform future intervention strategies in Latin America;
• self-reported and objective information on physical activity and sedentary time.

## Graphical abstract

**Figure:**
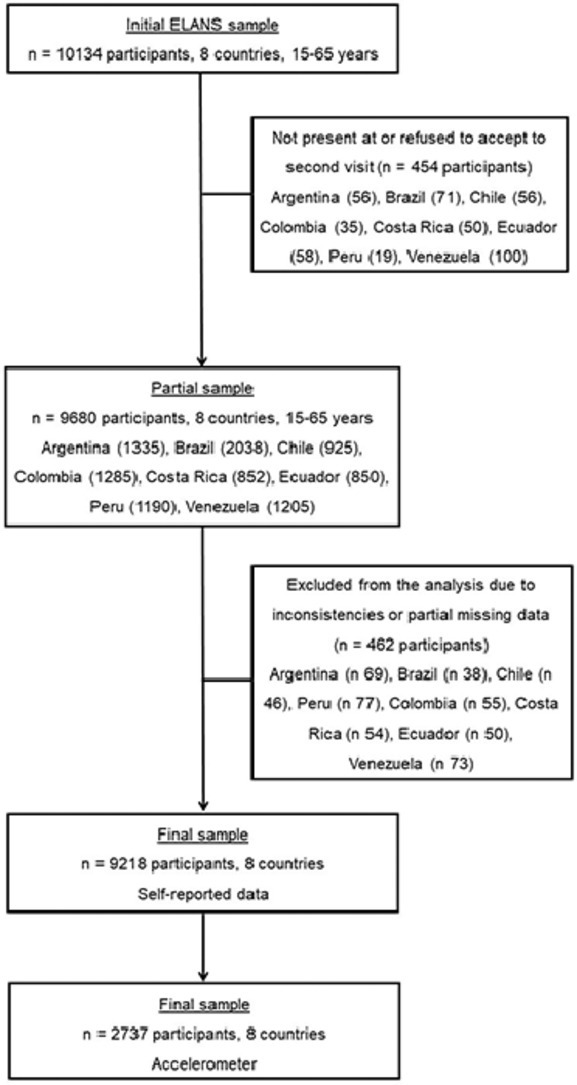

**Table:** Specifications Table

Subject Area	Medicine and dentistry.
More specific subject area	Measurement of physical activity and sedentary behaviours in the Latin American Study of Nutrition and Health.
Method name	ELANS study
Name and reference of original method	M. Fisberg, I. Kovalskys, G. Gomez, A. Rigotti, L.Y. Cortes, M. Herrera-Cuenca, et al., Latin American Study of Nutrition and Health (ELANS): rationale and study design. BMC Public Health. 16 (1) (2016) 93, doi: 10.1186/s12889–016–2765-y.
Resource availability	N/A

## Method details

### ELANS study

The Latin American Study of Nutrition and Health (*Estudio Latinoamericano de Nutrición y Salud*; ELANS) is a cross-sectional study, performed in nationally representative urban samples (persons aged 15–65 years) from eight countries: Argentina, Brazil, Chile, Colombia, Costa Rica, Ecuador, Peru and Venezuela [1]. Data collection took place in 2014 and 2015. The majority of the eight countries included in ELANS have at least 64–92% of the population living in urban areas.

The sample design for ELANS was complex and multistage. It was stratified by conglomerates (cities or agglomerations of cities), with random selection of Primary Sampling Units (PSUs) and Secondary Sampling Units (SSUs). The PSUs were areas (e.g. counties, municipalities, neighbourhoods, residential areas) within each selected city in each country. An “n” size proportional to the population weight was used for the selection of PSUs. In this case, a simple random sampling of “n” with replacement was performed to adhere to the principle of statistical independence of the selection of the areas included in the PSU sample. For these random selections, the probability proportional to size (PPS) method was applied. The SSU was the census radius or smallest unit of urban population data or sampling point, according to the available cartographic division. Thus, within each of the areas included in the PSU allocation, a representative sample of SSUs was randomly selected using the PPS method.

For the selection of households within SSUs, we performed a four-stage, systematic randomization by establishing a selection interval (k). In the first stage, the total urban population was used to proportionally define the main regions and to select cities representing each region, including key cities and other representative cities in the region, using a random method and sampling criteria, while attempting to cover the determined urban population. In the second stage, the sampling points (survey tracts) of each city were randomly designated. In the third stage, clusters of households were selected from each sampling unit. Addresses were chosen systematically using standard random route procedures, beginning with an initial address selected at random. The households were designated with three systematic jumps; that is, a given household was selected by randomly picking the first home and subsequently skipping three households. In the fourth and final stage, the designated respondent within each household was selected using the birthday method (half chosen using the next birthday; the other half using the last birthday).

The required sampling size for sufficient precision for key survey estimates was estimated by setting a confidence level of 95%, a maximum sampling error of 3.5%, and a survey design effect of 1.75 [2]. Table 1 shows the characteristics of the archived sample per sex, age group, and socioeconomic level. Persons excluded from the study were: pregnant and lactating women, with physical or mental disabilities, residents in unfamiliar residential environments, illiterate, and those unable to give consent. Considering the puberty that occurs during adolescence as a phase with many biological and physiological changes, the inclusion of adolescents under 15 years of age would require the evaluation of the pubertal stage, which is performed through the evaluation of the breasts, genitals and pubic hair [3]. This was considered to be not feasible. Thus, to ensure that puberty was achieved, only adolescents aged 15 years and older were eligible to participate in ELANS. Additional information on the ELANS design and sample size calculations are available elsewhere [1].

**Table 1:** Baseline characteristics of the sample assessed by sex, age-group, socioeconomic level and education level.

Country	*N*	Sex (%)		Age group (%)				Socioeconomic level (%)			Educational level (%)		
		Male	Female	15–19 y	20–34 y	35–49 y	50–65 y	Low	Medium	High	Low	Medium	High
Argentina	1266	48.6	51.4	13.2	33.3	30.1	23.4	45.7	46.8	7.4	65.7	25.9	8.4
Brazil	2000	48.2	51.8	12.0	37.0	30.6	20.5	45.3	46.0	8.8	48.1	43.4	8.5
Chile	879	49.3	50.7	10.4	34.9	29.8	24.9	45.4	38.5	16.1	61.1	22.0	17.0
Colombia	1230	48.2	51.8	11.3	36.7	29.1	22.9	52.8	42.0	5.1	63.2	23.8	13.0
Costa Rica	798	47.4	52.6	13.9	40.0	27.7	18.4	29.6	53.0	17.4	71.2	17.0	11.8
Ecuador	800	49.6	50.4	16.0	39.5	27.8	16.8	49.9	37.1	13.0	83.0	10.5	6.5
Peru	1113	47.0	53.0	14.8	41.3	26.4	17.4	47.9	31.9	20.2	23.1	67.1	9.8
Venezuela	1132	50.0	50.0	13.6	37.9	29.2	19.3	77.9	16.6	5.5	69.4	12.2	18.4

**Overall**	**9218**	**47.9%**	**52.1%**	**13.3%**	**37.7%**	**28.5%**	**20.5%**	**52.0%**	**38.4%**	**9.5%**	**61.3%**	**29.3%**	**9.5%**

The ELANS protocol was designed to collect data at the individual level using questionnaires (sociodemographic, dietary intake, and physical activity) and objective measurements (accelerometry and anthropometry). The ELANS protocol was approved by the Western Institutional Review Board (#20140605) and is registered with Clinicaltrials.gov (#NCT02226627). Local ethics committees also approved the protocol within each participating country. All participants signed the consent form before participating in the study.

### Governance, study management and quality control

A standard study protocol was distributed for implementation in each country. Principal Investigators (PIs, from Argentina and Brazil), and a co-chair (from Costa Rica), were responsible for overall coordination of the study. Local PIs managed each study site; these were responsible for all local study aspects including protocol development, regulatory oversight, training personnel, quality control, and logistics. Training systematized for all PIs and researchers was conducted before data collection. All researchers involved in ELANS underwent training and adhered to a single study protocol for implementation of fieldwork, data collection and management, and data quality control. Furthermore, implementation of the protocol included web-based training modules and regional direct training meetings. Quality control was monitored during data collection in all countries through verification of source documents and data entry errors. Data entry errors, and any other problems encountered during the verification of the source data, were discussed with the PIs.

### Assessment of physical activity

Physical activity can be assessed by several indicators, incorporating a spectrum of outcomes, including frequency (e.g. how often activities are performed), duration (how long activities are performed for), and intensity (i.e. the rate at which the activity is being performed or the magnitude of the effort required to perform an activity; usually classified as ‘light’, ‘moderate’, or ‘vigorous’). Self-report questionnaires facilitate data collection on specific types of activity (e.g. walking, cycling) and domains (e.g. active transportation, leisure-time) of physical activity and sedentary behaviour (e.g. time spent setting) [4]. However, questionnaires provide only a subjective estimate of physical activity and sedentary time. Reliance on respondent recall is associated with potential measurement error, and self-reports may be subject to social desirability bias. On the other hand, objective methods for evaluating physical activity, such as the use of accelerometers, obtain information such as the duration and the intensity of physical activity performed without the need for respondent recall or judgement. Placing accelerometers within large-scale national surveys requires a combination of financial resources and technological expertise that has so far challenged physical activity researchers based in low- and middle-income countries. As a result, Latin American researchers have typically relied on self-reported questionnaires to quantify physical activity and sedentary time both overall and across population subgroups (e.g. by age, sex, and socioeconomic status) [5].

**Fig. 1:**
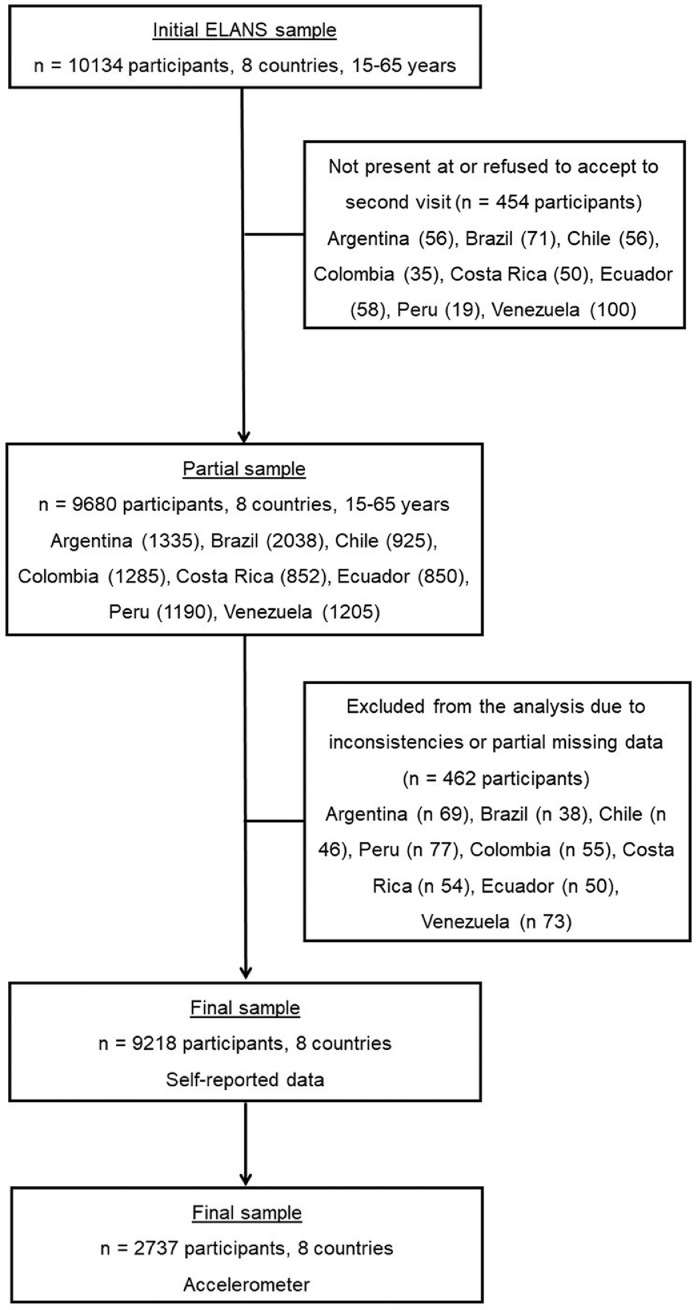
Flow-chart of the process to obtain the final sample.

The physical activity protocol used in ELANS includes self-reported data collected by questionnaires and objective data collected by triaxial accelerometers (Fig. 1). Data was collected via two home visits. At the first visit, a subsample of the designated respondents received instructions regarding the use of an accelerometer along with a diary to be filled out for seven consecutive days. For the participants who were given accelerometers, the second visit occurred eight days after the first contact. The second visit occurred four days after the first visit for those participants not in the accelerometry sub-sample. The second visit included the administration of the physical activity questionnaire, and the retrieval of the accelerometer and diary. Details of the mode of assessment of physical activity and the total number of participants by country, region and city are provided in Table 2.

**Table 2:** Sample distribution by region of countries, selected cities and proportion of total sample with self-reported physical activity data and valid accelerometer data.

Region	City	Urban population (15–65 years old / per city)	n total by city	n Self-reported data	Response rate of self-reported/ n total	N valid accelerometer data	% of total with valid accelerometer data
**Argentina**							
**Gran Buenos Aires**	Ciudad de Bs As + Gran Bs As	12,806	359	359	100%	80	22.3%
	Buenos Aires	2891	109	109	100%	26	23.9%
**Pampeana**	Gran Córdoba	1368	159	159	100%	35	22.0%
	Gran Rosario	1161	120	120	100%	28	23.3%
	Mar del Plata	593	61	60	98.3%	14	23.0%
	Rio Cuarto	163	35	35	100%	6	17.1%
**Cuyo**	Gran Mendoza	848	102	102	100%	28	27.5%
**Noroeste**	Gran S. Miguel de Tucumán	738	97	97	100%	33	34.0%
	Gran Salta	539	41	41	100%	10	24.4%
**Nordeste**	Corrientes	386	70	70	100%	9	12.9%
	Resistencia	356	69	69	100%	18	26.1%
**Sur**	Neuquén-Cipoletti (RN)	341	44	43	97.7%	9	20.5%
**Total**		**19,303**	**1266**	**1264**	**99.7%**	**296**	**23.4%**

**Brazil**							
**Norte**	Rio Branco	209	25	25	100%	8	32.0%
	Belém	991	112	112	100%	30	26.8%
**Nordeste**	Salvador	1972	129	129	100%	29	22.5%
	Fortaleza	1750	141	141	100%	43	30.5%
	João Pessoa	515	42	42	100%	18	42.9%
	Teresina	549	40	40	100%	17	42.5%
	Jaboatão dos Guararapes	447	30	30	100%	12	40.0%
**Sudeste**	Belo Horizonte	1735	114	114	100%	33	28.9%
	Rio de janeiro	4481	295	295	100%	64	21.7%
	São Paulo	8004	469	468	99.8%	145	30.9%
	Uberlândia	429	31	31	100%	10	32.3%
	Campinas	774	58	58	100%	14	24.1%
	Santos	293	17	17	100%	3	17.6%
	Vila Velha	298	16	16	100%	6	37.5%
	São Gonçalo	719	43	43	100%	13	30.2%
	Niterói	351	23	23	100%	4	17.4%
	São Bernardo do Campo	552	26	26	100%	8	30.8%
	Carapicuíba	263	24	24	100%	6	25.0%
**Sul**	Curitiba	1280	111	111	100%	38	34.2%
	Porto Alegre	1008	73	73	100%	25	34.2%
	Pelotas	216	13	10	76.9%	4	30.8%
**Centro-Oeste**	Brasília	1785	127	127	100%	30	23.6%
	Campo Grande	555	41	41	100%	4	9.8%
**Total**		**50,399**	**2000**	**1996**	**98.9%**	**564**	**28.2%**
**Chile**							
II. de Antofagasta	Antofagasta	289	35	35	100%	3	8.6%
V. de Valparaíso	Valparaíso	274	50	50	100%	13	26.0%
	Viña del Mar	285	58	58	100%	19	32.8%
VII. Del Maule	Talca	201	61	61	100%	20	32.8%
VIII. del Biobío	Concepción	217	66	66	100%	28	42.4%
	Talcahuano	163	64	64	100%	28	43.8%
XIII. Metropolitana	Gran Santiago	6061	429	429	100%	147	34.3%
IX. Araucanía	Temuco	246	63	62	98.4%	21	33.3%
X. Lagos	Puerto Montt	174	53	52	98.1%	18	34.0%
**Total**		**7914**	**879**	**877**	**99.6%**	**297**	**33.8%**

**Colombia**							
Andina	Bogotá D.C.	7776	274	273	99.6%	101	36.9%
	Medellín	2.375	124	123	99.2%	36	29.0%
	Cúcuta	615	95	93	97.9%	26	27.4%
	Bucaramanga	519	95	93	97.9%	35	36.8%
	Ibagué	510	85	85	100%	10	11.8%
	Pereira	388	85	85	100%	21	24.7%
Pacífico	Cali	2279	120	118	98.3%	16	13.3%
	Pasto	234	44	43	97.7%	5	11.4%
	Popayan	194	43	43	100%	7	16.3%
Caribe	Barranquilla	1202	151	151	100%	52	34.4%
	Cartagena	923	114	114	100%	30	26.3%
**Total**		**17,020**	**1230**	**1221**	**99.1%**	**339**	**27.6%**

**Costa Rica**							
San José	San Jose	1213	309	308	99.7%	96	31.1%
Alajuela	Alajuela	515	131	130	99.2%	42	32.1%
Cartago	Cartago	404	102	101	99.0%	44	43.1%
Heredia	Heredia	372	95	94	98.9%	34	35.8%
Guanacaste	Liberia	180	46	46	100%	17	37.0%
Puntarenas	Puntarenas	224	57	57	100%	23	40.4%
Limón	Limón	218	58	58	100%	17	29.3%
**Total**	**3130**	**798**	**794**	**99.5%**	**273**	**34.2%**	
**Ecuador**							

Costa	Guayaquil	2278	337	337	100%	115	34.1%
	Manchala	231	37	37	100%	8	21.6%
	Portoviejo	206	31	31	100%	10	32.3%
	Manta	217	35	35	100%	10	28.6%
Sierra	Quito	1607	241	239	99.2%	91	37.8%
	Cuenca	329	47	47	100%	15	31.9%
	Ambato	165	27	27	100%	6	22.2%
	Loja	170	22	22	100%	7	31.8%
	Ibarra	131	23	23	100%	6	26.1%
**Total**		**5339**	**800**	**798**	**99.8%**	**268**	**33.5%**

**Peru**							
Lima	Lima Metro	9740	483	481	99.6%	145	30.0%
Costa Norte	Trujillo	783	92	92	100%	27	29.3%
	Chiclayo	565	62	62	100%	22	35.5%
	Piura	430	57	57	100%	20	35.1%
Costa Sur	Arequipa	859	63	63	100%	18	28.6%
Sierra Centro	Huancayo	347	71	71	100%	23	32.4%
Sierra Sur	Cusco	417	93	93	100%	31	33.3%
	Juliaca	261	64	64	100%	19	29.7%
Oriente / Selva	Iquitos	423	83	83	100%	16	19.3%
	Pucallpa	229	45	40	88.9%	12	26.7%
**Total**		**14,058**	**1113**	**1106**	**98.5%**	**333**	**29.9%**

**Venezuela**							
Capital	Gran Caracas	6967	228	228	100%	84	36.8%
Oriental	Barcelona	326	119	119	100%	47	39.5%
Los Llanos	Guanare	160	53	53	100%	20	37.7%
	Barinas	970	101	101	100%	36	35.6%
Central	Valencia	1396	112	112	100%	28	25.0%
	Barquisimeto	881	112	112	100%	23	20.5%
	Maracay	955	74	74	100%	25	33.8%
Guayana	Ciudad Bolívar	407	63	63	100%	23	36.5%
Los Andes	San Cristóbal	282	60	59	98.3%	21	35.0%
	Mérida	248	42	42	100%	12	28.6%
Occidental	Maracaibo	2212	168	168	100%	48	28.6%
**Total**		**14,808**	**1132**	**1131**	**99.8%**	**367**	**32.4%**

**Overall**			**9218**	**9187**	**99.4%**	**2737**	**29.7%**

### Self-reported data

Physical activity and sedentary time were assessed in the second home visit using a Spanish language long-form, last 7-day, self-administered version of the International Physical Activity Questionnaire (IPAQ) [6]. We adapted the IPAQ by using only the questions that covered the active transportation and leisure-time domains, as well as those on sedentary time. Self-reported data was administered to the full sample (Table 1). Details on the development, reliability, and validity of the IPAQ are available elsewhere [6]. Only the active transportation and leisure-time physical activity sections were included due to the greater relevance of these domains for guiding public health policies and programmes [7] and the relatively low validity of the IPAQ items on occupational and home-based physical activity questions in Latin American urban settings [8].

The questions used in the ELANS study assessed physical activity in the active transportation and leisure-time domains using items on the frequency and duration (bouts of at least 10 min) of moderate-and-vigorous physical activity, as well as on walking (for leisure and active transportation), and the amount of cycling undertaken for the purposes of active transportation. Three outcomes for each domain were calculated to reflect the complexity of physical activity patterns.

• For the active transportation domain, the following questions were asked: (i) “During the last 7 days, did you walk or use a bicycle (pedal cycle) for at least 10 min continuously to get to and from places?” (Yes, No); (ii) “During the last 7 days, on how many days did you walk or ride a bicycle for at least 10 min at a time to go from place to place?”; (iii) “How much time did you usually spend on one of those days to bicycle or walk from place to place?” These questions were asked separately for walking and cycling.
• In relation to leisure (i.e. non travel-based) physical activity, the following questions were asked: (i) “During the last 7 days, did you walk, or do any moderate or vigorous physical activity for at least 10 min continuously?” (Yes, No); (ii) “During the last 7 days, on how many days did you walk, or do moderate or vigorous physical activity for at least 10 min at a time in your leisure time?”; (iii) “How much time do you spend walking, or doing moderate or vigorous physical activity in your leisure time?” Questions were asked separately for walking, moderate-intensity, and vigorous-intensity activities.

Thus, questionnaire data on physical activity was reported as minutes/day of walking, moderate, and vigorous physical activity. Data were analyzed in accordance with the IPAQ scoring protocol (https://sites.google.com/site/theipaq/scoring-protocol). The metabolic equivalents (METs) – by minutes/day and minutes/week (MET-min/day and MET-min/week, respectively) – in each physical activity was calculated in accordance with the Compendium of Physical Activities [9], [10]. Although walking is assigned as a moderate-intensity activity by MET value, as described above, walking was assessed separately from other moderate-intensity activities. The total time (minutes/week) and time spent in each physical activity mode (i.e. active transportation and leisure-time) were estimated. We analyzed total physical activity as the sum of active transport (walking and cycling) and leisure-time (walking, moderate, and vigorous) activities. In addition, we assessed domain-specific activity (active transportation and leisure-time). For total activity, participants were categorized as “meeting” or “not meeting” the World Health Organization (WHO) weekly recommendations of physical activity of at least 150 min of moderate-to-vigorous intensity [11].

In addition, we used two items capturing sedentary time. Participants were asked to estimate the total number of minutes per day spent sitting at work, at home, and during leisure-time separately for a weekday and a weekend day [12]. We summed the two weekday and weekend day items to calculate the overall daily sedentary time (weekday sitting minutes*5 weekdays + weekend day sitting minutes*2 weekend days)/7.

### Triaxial accelerometers

In the first home visit, the subsample of designated participants received the accelerometers with the respective instructions, including a diary to be completed for the following seven consecutive days. The accelerometers were retrieved on the second home visit. Accelerometer data were collected for 40% of the sample who were randomly selected to fill quotas by sex, age, and socioeconomic status, thereby ensuring a representative sample across these dimensions. For logistical and financial reasons, efforts were made to ensure that a range of 23.4–34.2% of each sample wore the accelerometer on all seven days (Table 1). Table 3 shows the overall and country-specific number of participants who were administered accelerometers and the proportions with valid data (i.e. minimum amount of accelerometer data). As Table 3 shows, the proportion of participants in the sub-sample with valid accelerometer data was highest and lowest in Chile (77.6%) and in Argentina (41.8%), respectively.

**Table 3:** Number of ELANS participants with accelerometers and the proportion with valid data.

Country	Total accelerometers distributed to participants: *n*	Total not valid accelerometer data: *n* (%)	Valid accelerometer data: *n* (%)
Argentina	509	213 (41.8)	296 (58.2)
Brazil	887	323 (36.4)	564 (63.6)
Chile	383	86 (22.4)	297 (77.6)
Colombia	577	238 (41.2)	339 (58.8)
Costa Rica	362	89 (24.6)	273 (75.4)
Ecuador	437	169 (38.7)	268 (61.3)
Peru	489	156 (31.9)	333 (68.1)
Venezuela	474	107 (22.6)	367 (77.4)
**Overall**	4118	1381 (32.4)	2737 (67.5)

Physical activity at different intensities (light, moderate, vigorous, and moderate-to-vigorous), number of steps, and the amount of time spent sedentary were measured using a triaxial accelerometer (model GT3X+, ActiGraph, Pensacola, FL, USA). The triaxial accelerometer is a small and light-weight device (4.6 cm × 3.3 cm × 1.5 cm, 19 g) designed to detect accelerations ranging in magnitude from ±6.00 G's with a frequency response of 0.25 to 2.50 Hertz and which converts the signal to numeric values known as activity counts [13]. These parameters detect natural movement of the body and filter out high frequency movement, such as vibrations [13]. The reliability and validity of accelerometers has been documented extensively in laboratory and in free-living conditions [14], [15], [16], [17].

Consistent with other studies using this device, we classified <100 activity counts/minutes (hereafter referred to as counts/min) as time spent sedentary [18]; 101–1951 counts/min as light-intensity activity; 1952–5724 counts/min as moderate; 5725–9498 counts/min as vigorous; and >9498 counts/min as very vigorous. Time spent in moderate-to-vigorous physical activity was classed as ≥1952 counts/min [14]. The cutoff points published previously were used to estimate METs [14].

An instruction sheet for the accelerometer, which contained a brief description of the device, details of how to wear it, and contact information, was left at the participant's home at the time of the first interview visit. Participants were asked to wear the accelerometer on an elasticized belt at hip level on the right mid-axillary line for seven days. Participants were asked to wear the device while awake and to remove it when sleeping, showering or swimming. Participants were encouraged to wear the accelerometer for at least 12 h/day for at least seven days. The minimum amount of accelerometer data we considered acceptable for analytical purposes (i.e., considered as valid data for our purposes) was five days (including at least one weekend day) with at least ten hours/day of wear time following the removal of sleep time [19], [20]. Data were processed using ActiLife software (V6.0; ActiGraph, Pensacola, FL). Data were collected at a sampling rate of 30 Hz and downloaded in epochs of 60 s. Data points were classified as nonwear time if the intervals showed at least 60 consecutive minutes of zero activity counts [21].

Two researchers received the raw data files through a cloud storage system. These researchers were responsible for performing data quality control and data validation with the help of the logbook information. Among the lessons learned during the fieldwork of our study, we feel it is important to have a team specifically trained to schedule and download the accelerometer data in order to avoid possible errors. In addition, managing the use of the devices by the participants, through telephone contacts and/or text messages helps in reducing the quantity of data that is not valid (e.g. for reasons of forgetfulness or insufficient hours of use per day).

### Analysis plan

Our analyses will be country-specific, and will involve describing the prevalence of physical inactivity (i.e. not meeting the WHO guidelines), and will summarize the amount of time spent sedentary, in different domains (leisure-time, active transportation) as well as overall, and by the intensity of physical activity (light, moderate, vigorous, very vigorous, and moderate-to-vigorous) (Table 4). Furthermore, the summary measures of physical activity and sedentary time will be presented by key socio-demographic variables (sex, age, socioeconomic status). Given that the aim of the ELANS study is to predict levels of physical activity and sedentary time, generalized linear and nonlinear models will be employed to investigate associations between physical activity and sedentary time and their correlates. Multilevel models taking into account the hierarchical nature of the ELANS data (i.e. participants within cities and within regions) will be used for all analyses. Statistical tests will be two-tailed and performed at the 5% significance level.

**Table 4:** Summary of method of assessment (self-reported data and accelerometer) and outcome.

Method of assessment	Outcome
Self-reported data	Physical activity (min/day, min/week, MET-min/day, or MET-min/week) • Leisure-time (walk, moderate, and vigorous physical activity) • Active transportation (walk, bicycle, and total physical activity) • Total • MET-min/day • MET-min/week • “Meeting” or “not meeting” the WHO recommendations Sitting time (min/day, and min/week) • Weekdays • Weekend days • Total

Accelerometer (model GT3X+, ActiGraph)	Physical activity intensity (min/day) • Light • Moderate • Vigorous • Very vigorous • Moderate–to–vigorous Counts Steps/day| Sedentary behaviours (min/day) Moderate-to-vigorous physical activity bouts per day METs Physical activity on weekdays and weekend

## Declaration of Competing Interest

The authors declare that they have no known competing financial interests or personal relationships that could have appeared to influence the work reported in this paper.
